# Catheter-Associated Urinary Tract Infections (CAUTI) and Central Line-Associated Bloodstream Infections (CLABSI) in Critical Care Units of a Tertiary Care Hospital: A Retrospective Surveillance Study

**DOI:** 10.7759/cureus.112623

**Published:** 2026-07-13

**Authors:** Amber Prasad, Minakshi Singh, Puneet K Gupta, Ankit Agarwal, Prasan K Panda, Gaurav Jain, Balram Omar, Pratima Gupta

**Affiliations:** 1 Microbiology, All India Institute of Medical Sciences Rishikesh, Rishikesh, IND; 2 Microbiology, Himalayan Institute of Medical Sciences, Swami Rama Himalayan University, Dehradun, IND; 3 Microbiology, All India Institute of Medical Sciences Bilaspur, Bilaspur, IND; 4 Anesthesiology, All India Institute of Medical Sciences Rishikesh, Rishikesh, IND; 5 Medicine, All India Institute of Medical Sciences Rishikesh, Rishikesh, IND

**Keywords:** catheter-associated urinary tract infection, cauti, central line-associated bloodstream infection, clabsi, covid-19, critical care, device utilisation ratio, hand hygiene

## Abstract

Background: Catheter-associated urinary tract infections (CAUTI) and central line-associated bloodstream infections (CLABSI) are important preventable device-associated healthcare-related infections in critical care settings and are associated with prolonged hospitalization, adverse outcomes, and increased treatment burden. This study evaluated the incidence and trends of these infections in the critical care units of a tertiary care hospital during the study period.

Methodology: This was a retrospective analysis of routinely collected surveillance records from the critical care units of a tertiary care teaching hospital from July 2019 to December 2020. Standard surveillance definitions were applied throughout. Data on patient-days, device-days, CAUTI events, CLABSI events, device utilization, and hand hygiene compliance were collected and analyzed. Trends across the pre-pandemic and pandemic periods were compared, and the association between hand hygiene compliance and infection rates was assessed.

Results: During the study period, 30,263 patient-days, 16,943 urinary catheter-days, and 11,891 central line-days were recorded. A total of 43 CAUTI events and 138 CLABSI events were identified. Infection rates varied across months and units, with higher burdens observed in some adult critical care areas and among lower birth-weight groups in the neonatal intensive care unit. CLABSI rates increased substantially during the pandemic period compared with the pre-pandemic period, while CAUTI rates also showed variation over time. Hand hygiene compliance remained low during the surveillance period.

Conclusions: Device-associated infection rates remained high in the critical care units studied and worsened during the pandemic period, particularly for CLABSI. Strengthening device-care practices, maintaining surveillance systems, and improving hand hygiene compliance remain essential to reducing infection burden in tertiary critical care settings.

## Introduction

Healthcare-associated infections (HAIs) represent a major global patient safety challenge, with catheter-associated urinary tract infections (CAUTI) and central line-associated bloodstream infections (CLABSI) consistently ranked among the most common, most costly, and most preventable [1,2]. CAUTI is the single most common HAI globally, accounting for approximately 30%-40% of all hospital-acquired infections [3], while CLABSI carries the highest attributable mortality of all device-associated infections, increasing intensive care unit (ICU) mortality by 12%-25% and prolonging hospital stay by 7-21 days [4]. Together, they impose an enormous clinical and economic burden, disproportionately heavy in low- and middle-income countries (LMICs) such as India [5].

Data from the International Nosocomial Infection Control Consortium (INICC) across 20 Indian cities documented a pooled CLABSI rate of 7.92 per 1,000 central line (CL)-days, approximately eight times the National Healthcare Safety Network (NHSN) benchmark of 0.9 per 1,000 for comparable US medical ICUs [6]. CAUTI rates in Indian tertiary centers have ranged from 1.6 to 8.9 per 1,000 catheter-days [7]. Contributing factors include high device utilization ratios (DURs), overcrowded ICUs, low nurse-to-patient staffing ratios, limited trained infection prevention and control (IPC) personnel, and persistently suboptimal hand hygiene (HH) compliance [8]. Despite national IPC guidelines and hospital quality frameworks that mandate monthly CAUTI and CLABSI surveillance using device-day denominators, published longitudinal HAI data from Indian tertiary hospitals remain limited.

The coronavirus disease 2019 (COVID-19) pandemic profoundly disrupted IPC in 2020. Rapid conversion of ward areas into ICUs, redeployment of trained infection control nurses, critical shortages of personal protective equipment (PPE), and the high device demands of COVID-19 critical illness collectively drove up device-related HAI rates internationally [9,10]. Multiple studies confirmed significant pandemic-related increases in CLABSI and CAUTI rates even in countries with stronger IPC infrastructure than India [9,10], implying a greater impact in resource-limited settings.

All India Institute of Medical Sciences (AIIMS) Rishikesh is a 960-bed apex central government teaching hospital and the primary critical care referral center for approximately 15 million people across Uttarakhand, Himachal Pradesh, and western Uttar Pradesh. From July 2020, the institution operated four dedicated COVID-19 ICUs alongside its established critical care units (CCUs). This study presents a comprehensive retrospective analysis of CAUTI and CLABSI surveillance data from the Hospital Infection Control Committee (HICC) covering the full available 18-month period (July 2019 to December 2020), with the primary objective of determining the incidence and temporal trends of CAUTI and CLABSI in CCUs using standardized NHSN surveillance definitions. Secondary objectives were to compare infection rates before and during the COVID-19 pandemic, identify high-risk clinical units, evaluate DURs, assess CLABSI rates according to neonatal birth-weight categories, and examine the relationship between HH compliance and device-associated infection rates.

## Materials and methods

Study design and setting

This was a retrospective descriptive surveillance study using routinely collected, de-identified HICC institutional records. AIIMS Rishikesh is a 960-bed apex tertiary teaching hospital under the Ministry of Health and Family Welfare, Government of India. The hospital's critical care infrastructure comprises a respiratory ICU (RICU), CCUs on the fourth and sixth floors (CCU-4 and CCU-6, respectively), a medicine high-dependency unit (medicine HDU), a medicine allied ICU, a neurosurgery ward, a neurosurgery HDU, an HDU and stroke unit, a pediatric medicine HDU, a pediatric surgery ICU, and a neonatal ICU (NICU). From July 2020, four dedicated COVID-19 ICUs (COVID ICU-1, ICU-2, ICU-3, and ICU-4) were additionally operational, bringing the total number of study units to 14.

Study population and eligibility criteria

All patients admitted to the above-mentioned CCUs and HDUs during the study period (July 2019 to December 2020) and exposed to an indwelling urinary catheter (Foley catheter) or central vascular catheter for more than two consecutive calendar days were included in the surveillance denominator. No individual patient-level exclusion criteria were applied, as this was a unit-level aggregate surveillance study. Patients transferred between units within the hospital were counted in the unit where the device-associated infection was identified. Pediatric and neonatal patients in the pediatric medicine HDU, pediatric surgery ICU, and NICU were included; NICU patients were further stratified by five birth-weight categories: extremely low birth weight (ELBW, <750 g), very low birth weight (VLBW, 751-1,000 g), low birth weight (LBW, 1,001-1,500 g), moderate low birth weight (MLBW, 1,501-2,500 g), and normal birth weight (NBW, >2,500 g).

Ethical considerations

Ethical approval was obtained from the Institutional Ethics Committee (IEC), AIIMS Rishikesh (Ref: AIIMS/IEC/21/24). As a retrospective analysis of anonymized aggregate HICC surveillance data with no individual patient identifiers, the study qualified for expedited review. Written informed consent was waived in accordance with the Indian Council of Medical Research (ICMR) guidelines for secondary data studies [11].

Data sources and study period

Four institutional HICC datasets were utilized, representing the complete available surveillance record: (i) a July 2019 baseline line-day dataset with department-level Foley-days, CL-days, CAUTI events, and CLABSI events; (ii) monthly unit-level HAI cumulative records for January to December 2020 (11 monthly datasets); (iii) monthly quality indicator returns from the HICC covering hospital-wide CAUTI rate, CLABSI/bloodstream infection (BSI) rate, and HH compliance from July 2019 to September 2020 (14 months); and (iv) a detailed CAUTI denominator dataset for December 2019 to June 2020.

The study period (July 2019 to December 2020) was stratified into three phases: Phase 1, pre-COVID-19 baseline (July 2019 to March 2020); Phase 2, pandemic surveillance gap (April to June 2020), during which routine HAI surveillance was suspended due to COVID-19 crisis response and no data were available; and Phase 3, COVID-19 era (July to December 2020), which encompassed the operation of dedicated COVID-19 ICUs and peak pandemic workload.

Case definitions

All infections were defined per the NHSN Patient Safety Component Manual [12]. CAUTI was defined as a symptomatic urinary tract infection (UTI) in a patient with an indwelling urinary catheter in place for more than two calendar days on the date of the event, or within one calendar day of its removal. CLABSI was defined as a primary BSI in a patient with a central vascular catheter in place for more than two calendar days on the date of the event and within one calendar day of its removal, with no other identified infection source. Device-associated BSI in HICC monthly returns includes both CLABSI and non-CLABSI CL-associated events.

Microbiological confirmation was performed in the Department of Microbiology according to standard laboratory protocols. Blood cultures were processed using automated blood culture systems, and urine cultures were performed using standard quantitative culture techniques. Organism identification and antimicrobial susceptibility testing were carried out using routine laboratory methods in accordance with contemporaneous Clinical and Laboratory Standards Institute (CLSI) recommendations. Final CAUTI and CLABSI diagnoses were established using NHSN surveillance definitions by the HICC.

Rate and ratio calculations

CAUTI rate (per 1,000 Foley-days) = (CAUTI events x 1,000)/indwelling urinary catheter-days. CLABSI/BSI rate (per 1,000 CL-days) = (CLABSI/BSI events x 1,000)/CL-days. DUR = device-days/patient-days. A Foley DUR > 0.50 and a CL DUR > 0.30 are considered high for medical ICUs per NHSN standards [13]. These DURs serve as proxies for the intensity of device use in a unit and are presented alongside infection rates to contextualize HAI risk.

Statistical analysis

Descriptive statistics, including mean, standard deviation (SD), range, and 95% confidence interval (CI; using the Poisson model for rates), were calculated for all infection rate variables. Monthly CAUTI and CLABSI rates were plotted as run charts to visualize temporal trends and identify shifts. Pre-COVID-19 (January to March 2020) versus COVID-19 era (July to December 2020) rates were compared using rate ratios (RRs) with 95% CI by the Poisson method; a two-sided p-value < 0.05 was considered statistically significant. The Spearman rank correlation coefficient (rho) was used to assess the monotonic relationship between monthly HH compliance (%) and concurrent CLABSI/BSI rates (per 1,000 CL-days); 14 paired monthly observations were available for this analysis. No sample size calculation was applicable as this was a retrospective analysis of an existing surveillance dataset covering all eligible unit-level admissions during the study period. All statistical analyses were performed using Python 3.12 (pandas version 2.0, scipy version 1.11; Python Software Foundation, Fredericksburg, VA, USA).

## Results

Study population and device exposure

Over the 18-month study period, a total of 30,263 patient-days, 16,943 Foley-days, and 11,891 CL-days were recorded across all CCUs and HDUs. The overall Foley DUR was 0.560, and the CL DUR was 0.393, substantially exceeding NHSN published median DURs for comparable US medical ICUs (Foley DUR approximately 0.37; CL DUR approximately 0.22) [13], indicating very high device utilization throughout. A marked contraction is visible in March 2020 (676 patient-days), corresponding to the national COVID-19 lockdown, followed by rapid expansion from July 2020 as COVID-19 ICUs opened, peaking in December 2020 (4,235 patient-days). The July 2019 data point (2,541 patient-days, 1,127 Foley-days, and 639 CL-days) is included in all cumulative analyses. Monthly patient-days, device-days, DURs, and infection events are presented in Table 1.

**Table 1:** Monthly patient-days, device-days, and utilization ratios -- all critical care and high-dependency areas (2019-2020) Aug-Nov 2019: unit-level line-day data not available; hospital-wide HICC rates and HH compliance recorded. Dec 2019: Foley-day denominator and CAUTI events from the CAUTI detailed dataset only. The total row includes July 2019, January-March 2020, and July-December 2020. *Statistical significance at p < 0.05. CL: central line; DUR: device utilization ratio; HH: hand hygiene; N/A: not collected; --: surveillance gap (COVID-19 pandemic response) or not reported; CAUTI: catheter-associated urinary tract infection; CLABSI: central line-associated bloodstream infection; HICC: Hospital Infection Control Committee; COVID-19: coronavirus disease 2019

Month/period	Patient-days	Foley-days	CL-days	Foley DUR	CL DUR	HH compliance (%)	CAUTI events	CLABSI events
Jul 2019	2,541	1,127	639	0.444	0.251	30.0	2	12
Aug 2019*	--	--	--	--	--	16.7	--	--
Sep 2019*	--	--	--	--	--	17.7	--	--
Oct 2019*	--	--	--	--	--	26.8	--	--
Nov 2019*	--	--	--	--	--	20.1	--	--
Dec 2019*	--	--	1,254	--	--	28.2	7	--
Jan 2020	2,785	1,623	981	0.583	0.352	24.7	3	3
Feb 2020	2,571	1,439	924	0.560	0.359	27.8	2	6
Mar 2020	676	311	329	0.460	0.487	22.7	1	2
Apr-Jun 2020	--	--	--	--	--	--	N/A	N/A
May 2020 (partial)	469	252	286	0.537	0.610	3.7	0	3
Jun 2020 (partial)	432	285	273	0.660	0.632	20.2	1	2
Jul 2020	2,178	1,258	880	0.578	0.404	15.6	3	20
Aug 2020	3,875	1,888	1,338	0.487	0.345	16.3	10	12
Sep 2020	3,259	2,213	1,673	0.679	0.513	30.3	3	21
Oct 2020	3,476	2,036	1,467	0.586	0.422	--	7	26
Nov 2020	3,766	2,134	1,480	0.567	0.393	--	6	15
Dec 2020	4,235	2,377	1,621	0.561	0.383	--	5	16
Total	30,263	16,943	11,891	0.560	0.393	--	43	138

**Figure 1:**
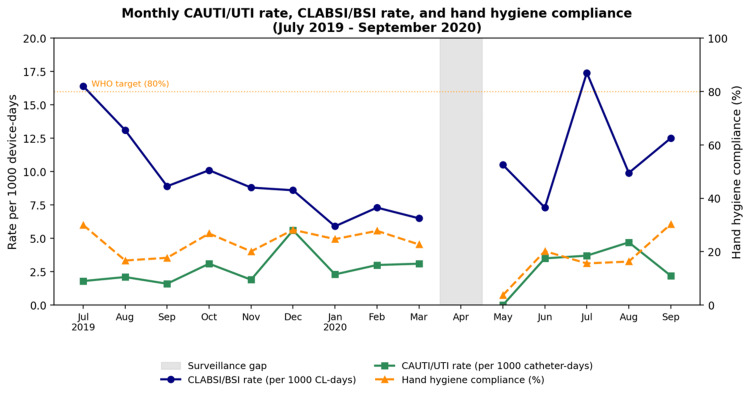
Monthly CAUTI/UTI and CLABSI/BSI rates with HH compliance (July 2019-September 2020, 14 reporting months) Monthly HICC-recorded CAUTI/UTI rate (per 1,000 catheter-days), CLABSI/BSI rate (per 1,000 CL-days), and HH compliance (%) from July 2019 to September 2020 (n = 14 reporting months). The shaded zone (April-June 2020) indicates the COVID-19-related surveillance suspension period. The CLABSI/BSI rate declined from 16.4 in July 2019 to 5.9 per 1,000 CL-days in January 2020 before surging to 17.4 per 1,000 CL-days in July 2020. HH compliance never exceeded 30.3% in any reporting month; the dotted line at 80% represents the WHO minimum HH compliance target. CAUTI: catheter-associated urinary tract infection; CLABSI: central line-associated bloodstream infection; BSI: bloodstream infection; UTI: urinary tract infection; HH: hand hygiene; CL: central line; WHO: World Health Organization; HICC: Hospital Infection Control Committee; COVID-19: coronavirus disease 2019

Temporal trends in hospital-wide HICC-recorded CAUTI/UTI rate, CLABSI/BSI rate, and HH compliance across the study period are shown in Figure 1. A steady decline in CLABSI/BSI rate from July 2019 (16.4 per 1,000 CL-days) to January 2020 (5.9 per 1,000) is evident, followed by a sharp surge in July 2020 (17.4 per 1,000) coinciding with the COVID-19 era. HH compliance reached its nadir of 3.7% in May 2020, the height of the pandemic lockdown.

CAUTI incidence and unit-level analysis

Over the full study period, 43 CAUTI events were recorded across 16,943 Foley-days (rate 2.54 per 1,000 Foley-days; 95% CI 1.84-3.42). Hospital-wide HICC-recorded CAUTI rates (14 months, July 2019 to September 2020) ranged from 0.0 to 5.6 per 1,000 catheter-days (mean 2.76 ± 1.45 SD). The December 2019 dataset confirmed seven CAUTI events across 1,254 Foley-days (rate 5.58 per 1,000), the highest single-month CAUTI rate in the detailed dataset. Unit-level CAUTI rates are shown in Table 2.

**Table 2:** Unit-specific CAUTI rates and Foley-day DURs -- full study period (July 2019 to December 2020) 90th-percentile benchmark for medical ICUs: >4.7 per 1,000 Foley-days; 75th-percentile: >1.4 per 1,000 Foley-days [13]. Statistical test: Poisson rate estimation; p < 0.05 considered significant. Units with fewer than 50 Foley-days excluded. *Statistical significance at p < 0.05. NHSN: National Healthcare Safety Network; HDU: high-dependency unit; ICU: intensive care unit; RICU: respiratory ICU; CCU: critical care unit; DUR: device utilization ratio; CAUTI: catheter-associated urinary tract infection; COVID: coronavirus disease

Unit	Patient-days	Foley-days	Foley DUR	CAUTI (n)	CAUTI rate*	vs. NHSN benchmark
Pediatric medicine HDU	1,840	908	0.493	6	6.61	Exceeds 90th percentile
CCU 4th floor	167	165	0.988	1	6.06	Exceeds 90th percentile
Neurosurgery ward	3,612	1,859	0.515	11	5.92	Exceeds 90th percentile
Medicine allied ICU	1,644	1,597	0.971	9	5.64	Exceeds 90th percentile
RICU	411	368	0.895	2	5.43	Exceeds 90th percentile
Neurosurgery HDU	293	293	1.000	1	3.41	Exceeds 75th percentile
Medicine HDU	1,872	1,140	0.609	3	2.63	Exceeds 75th percentile
CCU 6th floor	4,301	4,189	0.974	7	1.67	At/below benchmark
HDU and stroke	1,734	1,527	0.881	2	1.31	At/below benchmark
Neurosurgery	1,656	723	0.437	1	1.38	At/below benchmark
COVID ICU-1	1,751	1,191	0.680	0	0.00	Below benchmark
COVID ICU-3	860	695	0.808	0	0.00	Below benchmark

The pediatric medicine HDU had the highest CAUTI rate at 6.61 per 1,000 Foley-days (six events/908 Foley-days; DUR 0.493), followed by CCU fourth floor (6.06 per 1,000), neurosurgery ward (5.92 per 1,000; 11 events/1,859 Foley-days), medicine allied ICU (5.64 per 1,000; nine events/1,597 Foley-days), and RICU (5.43 per 1,000; two events/368 Foley-days; DUR 0.895). These units all had Foley DURs above 0.49, indicating near-universal catheterization. The CCU sixth floor had a notably lower CAUTI rate of 1.67 per 1,000 despite the highest Foley DUR (0.974), suggesting relatively effective catheter-care practices. Thirty-eight of 43 CAUTI events (88.4%) occurred in seven ICU/HDU areas.

CLABSI incidence and unit-level analysis

Over the full study period, 138 CLABSI events were recorded across 11,891 CL-days (rate 11.61 per 1,000 CL-days; 95% CI 9.74-13.68). Hospital-wide HICC-recorded BSI rate (July 2019 to September 2020; 14 months) ranged from 5.9 to 17.4 per 1,000 CL-days (mean 10.23 ± 3.62). A sustained 64% reduction was observed from July 2019 (16.4 per 1,000) to January 2020 (5.9 per 1,000), likely reflecting IPC bundle reinforcement and improved HH practices during that period. This improvement was completely reversed by July 2020 (17.4 per 1,000) following COVID-19 surge operations. Unit-level CLABSI data are shown in Table 3.

**Table 3:** Unit-specific CLABSI rates and CL-day DURs -- full study period (July 2019 to December 2020) 90th-percentile benchmark for medical ICUs: >2.9 per 1,000 CL-days; 75th-percentile: >0.9 per 1,000 CL-days [13]. Statistical test: Poisson rate estimation; p < 0.05 considered significant. All rate comparisons are per 1,000 CL-days. *Statistical significance at p < 0.05. NHSN: National Healthcare Safety Network; CL: central line; DUR: device utilization ratio; CLABSI: central line-associated bloodstream infection; ICU: intensive care unit; HDU: high-dependency unit; CCU: critical care unit; COVID: coronavirus disease

Unit	Patient-days	CL-days	CL DUR	CLABSI (n)	CLABSI rate*	vs. NHSN benchmark
CCU 4th floor	167	134	0.802	6	44.78	Far exceeds 90th percentile
Medicine allied D34 ICU	211	169	0.801	5	29.59	Far exceeds 90th percentile
Medicine allied ICU	1,644	1,225	0.745	24	19.59	Far exceeds 90th percentile
COVID ICU-1	1,751	890	0.508	16	17.98	Far exceeds 90th percentile
COVID ICU-3	860	561	0.652	8	14.26	Far exceeds 90th percentile
COVID ICU-2	2,306	623	0.270	8	12.84	Far exceeds 90th percentile
HDU and stroke	1,734	620	0.358	7	11.29	Far exceeds 90th percentile
Medicine HDU	1,872	627	0.335	6	9.57	Far exceeds 90th percentile
CCU 6th floor	4,301	3,939	0.916	32	8.12	Far exceeds 90th percentile
COVID ICU-4	2,252	283	0.126	2	7.07	Exceeds 75th percentile
Neurosurgery	1,656	297	0.179	2	6.73	Exceeds 75th percentile
Pediatric medicine HDU	1,840	409	0.222	2	4.89	Exceeds 75th percentile
Neurosurgery ward	3,612	921	0.255	4	4.34	Exceeds 75th percentile

CCU fourth floor had the highest CLABSI rate (44.78 per 1,000 CL-days; six events/134 CL-days; CL DUR 0.802). Medicine allied ICU had the highest absolute CLABSI count (24 events; 19.59 per 1,000; CL DUR 0.745). COVID ICU-1 recorded 16 CLABSI events (17.98 per 1,000), and COVID ICU-3 contributed eight events (14.26 per 1,000), confirming that newly commissioned COVID-19 ICUs were not protected from CLABSI risk. CCU sixth floor had the highest absolute CLABSI burden (32 events including July 2019; rate 8.12 per 1,000) owing to its large CL exposure (3,939 CL-days; CL DUR 0.916). All 13 units with sufficient CL-day denominators exceeded the NHSN 90th-percentile benchmark of 2.9 per 1,000 CL-days [13]. Across all units, 116 of 138 CLABSI events (84.1%) occurred in seven units: medicine allied ICU, CCU sixth floor, COVID ICU-1, CCU fourth floor, COVID ICU-3, COVID ICU-2, and HDU and stroke.

Pre-COVID-19 versus COVID-19 era comparison

Table 4 presents the pre-COVID-19 (January to March 2020) versus COVID-19 era (July to December 2020) comparison. CLABSI rate increased from 4.92 to 13.00 per 1,000 CL-days (RR 2.64; 95% CI 1.43-4.89; p < 0.01). CAUTI rate increased from 1.78 to 2.86 per 1,000 Foley-days (RR 1.61; 95% CI 0.68-3.82; p = NS). CL DUR increased from 0.370 to 0.407. Both COVID-19 ICUs (pooled rate 15.71 per 1,000 CL-days across 2,356 CL-days) and non-COVID-19 ICUs showed worsening CLABSI rates. Non-COVID-19 ICU rates rose from 3.25 per 1,000 CL-days in January 2020 to 18.17 per 1,000 in July 2020, confirming institution-wide IPC degradation rather than a problem confined to COVID-19-specific areas [9,10].

**Table 4:** Pre-COVID-19 versus COVID-19 era comparison of CAUTI and CLABSI metrics Statistical test: Poisson rate ratio test for infection rates; p < 0.05 considered significant. *Statistical significance at p < 0.05. NS: not significant (p > 0.05); CI: confidence interval; CL: central line; DUR: device utilization ratio; HH: hand hygiene; CAUTI: catheter-associated urinary tract infection; CLABSI: central line-associated bloodstream infection; COVID-19: coronavirus disease 2019

Parameter	Pre-COVID-19 (Jan-Mar 2020)	COVID-19 era (Jul-Dec 2020)	Rate ratio (95% CI)	Change
Patient-days	6,032	20,789	--	3.4-fold
Foley-days	3,373	11,906	--	3.5-fold
CL-days	2,234	8,459	--	3.8-fold
Foley DUR	0.559	0.573	--	+2.5%
CL DUR	0.370	0.407	--	+10.0%
CAUTI events	6	34	--	5.7-fold
CAUTI rate per 1,000 Foley-days	1.78	2.86	1.61 (0.68-3.82), NS	+60.7%
CLABSI events	11	110	--	10-fold
CLABSI rate per 1,000 CL-days	4.92	13.00	2.64 (1.43-4.89)*	+164%
Mean HH compliance (%)	24.7	20.7	--	-4.0 pp

NICU CLABSI: birth-weight-stratified analysis

Table 5 presents NICU CLABSI data, combining July 2019 and the full 2020 period across five birth-weight strata. CLABSI risk showed an inverse relationship with birth weight: ELBW neonates (<750 g) had the highest rate at 37.74 per 1,000 CL-days (CL DUR 0.636), consistent with the known vulnerability of extremely premature neonates due to immature immune function, fragile skin barrier, parenteral nutrition requirements, and prolonged CL use [14]. The 1,001-1,500 g (LBW) stratum recorded a rate of 25.42 per 1,000 CL-days despite a CL DUR of only 0.113, higher than the VLBW stratum (24.39 per 1,000; DUR 0.365). This paradox warrants investigation: possible explanations include prolonged CL dwell time, high rates of total parenteral nutrition (TPN), or clustering of events in a sub-period. All five strata exceeded the NHSN NICU very-low-birth-weight benchmark of 11.0 per 1,000 CL-days [13].

**Table 5:** NICU CLABSI rates stratified by BW category -- combined July 2019 and January to December 2020 data NHSN NICU VLBW benchmark: 11.0 per 1,000 CL-days [13]. Statistical test: Poisson rate estimation; p < 0.05 considered significant. All rates are per 1,000 CL-days. *Statistical significance at p < 0.05. NICU: neonatal ICU; BW: birth weight; CL: central line; DUR: device utilization ratio; CLABSI: central line-associated bloodstream infection; NHSN: National Healthcare Safety Network; ICU: intensive care unit

BW category	Abbreviation	Patient-days	CL-days	CL DUR	CLABSI events	CLABSI rate*
<750 g (extremely low BW)	ELBW	88	56	0.636	2	37.74
751-1,000 g (very low BW)	VLBW	337	123	0.365	3	24.39
1,001-1,500 g (low BW)	LBW	1,041	118	0.113	3	25.42
1,501-2,500 g (moderate low BW)	MLBW	1,600	91	0.057	1	10.99
>2,500 g (normal BW)	NBW	1,372	165	0.120	3	18.18

HH compliance

HH compliance (14 months, July 2019 to September 2020) ranged from 3.7% to 30.3% (mean 21.5% ± 7.1% SD). It never reached the WHO minimum target of 80% [15] in any single reporting month. Pre-COVID-19 mean was 23.9%, declining to 20.7% in the COVID-19 era. The nadir of 3.7% was recorded in May 2020 during the COVID-19 lockdown, suggesting a near-complete breakdown of routine IPC monitoring activities. Full monthly HH and infection rate data are shown in Table 6.

**Table 6:** Monthly HH compliance and HICC-recorded CAUTI and CLABSI/BSI rates (July 2019 to September 2020) *CAUTI/UTI rate per 1,000 catheter-days (HICC hospital-wide returns). ^+^CLABSI/BSI rate per 1,000 CL-days (HICC hospital-wide returns). Statistical test: Spearman rank correlation (rho) used to assess HH compliance vs. CLABSI/BSI rate; rho = -0.200, p = 0.493, n = 14 monthly pairs; p < 0.05 considered significant. N/A: not collected (surveillance gap); --: data not available for that month; HH: hand hygiene; CAUTI: catheter-associated urinary tract infection; CLABSI: central line-associated bloodstream infection; BSI: bloodstream infection; CL: central line; COVID-19: coronavirus disease 2019; HICC: Hospital Infection Control Committee; UTI: urinary tract infection

Month	CAUTI rate*	CLABSI/BSI rate^+^	HH compliance (%)	Directional observation	Foley-days	Phase
Jul 2019	1.8	16.4	30.0	HH moderate/BSI high	1,127	Pre-COVID-19
Aug 2019	2.1	13.1	16.7	HH low/BSI high	--	Pre-COVID-19
Sep 2019	1.6	8.9	17.7	HH low/BSI moderate	--	Pre-COVID-19
Oct 2019	3.1	10.1	26.8	HH moderate/BSI moderate	--	Pre-COVID-19
Nov 2019	1.9	8.8	20.1	--	--	Pre-COVID-19
Dec 2019	5.6	8.6	28.2	HH moderate/BSI moderate	1,254	Pre-COVID-19
Jan 2020	2.3	5.9	24.7	HH moderate/BSI nadir	1,284	Pre-COVID-19
Feb 2020	3.0	7.3	27.8	HH moderate/BSI low	1,329	Pre-COVID-19
Mar 2020	3.1	6.5	22.7	--	325	Pre-COVID-19
Apr 2020	N/A	N/A	N/A	Surveillance gap	--	Gap
May 2020	0.0	10.5	3.7	HH nadir/BSI elevated	252	Gap
Jun 2020	3.5	7.3	20.2	--	285	Gap
Jul 2020	3.7	17.4	15.6	HH low/BSI peak	1,258	COVID-19 era
Aug 2020	4.7	9.9	16.3	HH low/BSI high	1,888	COVID-19 era
Sep 2020	2.2	12.5	30.3	HH rising/BSI still high	2,213	COVID-19 era

The Spearman rank correlation between monthly HH compliance and concurrent BSI rates yielded rho = -0.200 (p = 0.493; n = 14). The direction is consistent with a protective effect of HH but did not reach statistical significance, expected given the small sample of 14 monthly pairs and substantial pandemic-related confounding. Three of the four months with the lowest HH compliance (≤16.7%) coincided with BSI rates ≥9.9 per 1,000 CL-days. The mechanistic relationship between HH failure and CLABSI is well established [16], and the non-significance here reflects the underpowered design rather than the absence of a true effect.

## Discussion

This 18-month retrospective surveillance study from AIIMS Rishikesh provides a comprehensive analysis of CAUTI and CLABSI across 14 distinct CCU and HDU types using NHSN device-day denominators -- the methodological standard enabling valid rate comparisons with published benchmarks. Five principal findings emerge: (i) CAUTI and CLABSI rates substantially exceeded NHSN benchmarks in virtually every unit throughout, (ii) a meaningful CLABSI improvement period was completely reversed by the COVID-19 pandemic, (iii) medicine allied ICU and CCU-4 are the highest-priority CLABSI intervention targets, (iv) all five NICU birth-weight strata exceeded published benchmarks, and (v) HH compliance remained persistently below WHO targets.

Benchmark context and comparison with Indian studies

Our overall CLABSI rate of 11.61 per 1,000 CL-days is consistent with INICC-India pooled data of 7.92-22 per 1,000 CL-days [6] and with published reports from other Indian tertiary centers: Mehta et al. reported a CLABSI rate of 8.3 per 1,000 CL-days from a tertiary care center in New Delhi [17]; Rosenthal et al. in the INICC 50-country report documented Indian ICU CLABSI rates of 6.7-16.3 per 1,000 CL-days [18]. However, our rate is 12.9 times the NHSN 50th-percentile benchmark of 0.9 per 1,000 and 4.0 times the 90th-percentile benchmark of 2.9 per 1,000 [13]. Every unit in our study with a sufficient denominator exceeded the NHSN 90th percentile, indicating a systemic infrastructure gap rather than unit-specific lapses.

Our CAUTI rate of 2.54 per 1,000 Foley-days is comparable with rates reported from Indian tertiary ICUs (1.6-8.9 per 1,000 catheter-days) [7] and with a multicenter INICC report across 50 countries (mean 4.7 per 1,000 catheter-days) [18]. However, five units in our study exceeded the NHSN 90th-percentile CAUTI benchmark of 4.7 per 1,000. High device DURs (Foley DUR 0.560; CL DUR 0.393), exceeding NHSN US medians by 54% and 85%, respectively [13], underscore device utilization reduction as an equally important prevention target alongside bundle implementation. In comparison, Jaggi and Sissodia, reporting from a tertiary hospital in India, demonstrated significant CAUTI rate reductions following implementation of a multimodal supervision program, achieving rates below 2.0 per 1,000 catheter-days [7].

The improvement period and COVID-19 reversal

The 64% reduction in hospital-wide BSI rate from July 2019 (16.4 per 1,000 CL-days) to January 2020 (5.9 per 1,000) is a clinically important finding demonstrating that sustained CLABSI improvement is achievable in this setting, likely reflecting IPC bundle reinforcement, CL insertion training, and improved HH auditing during the second half of 2019. This trajectory has been described in other Indian institutional studies following structured IPC program implementation [8].

The subsequent pandemic-related reversal -- with CLABSI rate rising to 17.4 per 1,000 in July 2020 -- mirrors international pandemic trends: Baker et al. documented a 47% national CLABSI rate increase in the United States in 2020 versus 2019 [10], while Fakih et al. reported nosocomial transmission amplification in European ICUs during peak pandemic waves [9]. Our 164% rate increase was substantially larger, likely reflecting the compounded burden of three months of complete surveillance disruption, rapid COVID-19 ICU commissioning without established IPC bundles, and redeployment of trained IPC nurses. Fakih et al. highlighted precisely this mechanism -- the "hardwiring" of prevention efforts being undone by pandemic-related distractions -- as a key modifiable driver of pandemic-era HAI increases [9].

Unit-specific and NICU insights

The extreme CLABSI rate in CCU-4 (44.78 per 1,000 CL-days; six events/134 CL-days) may reflect a cluster of events in a small unit associated with a specific procedural or environmental lapse and warrants a dedicated root-cause analysis. Medicine allied ICU's sustained high rate (19.59 per 1,000; 1,225 CL-days) represents a systemic problem: its CL DUR of 0.745 -- indicating three-quarters of patient-days involved a CL -- presents a major opportunity for device utilization reduction. The RICU's high CAUTI rate (5.43 per 1,000; Foley DUR 0.895) reflects near-universal catheterization of sedated, ventilated patients, while the pediatric medicine HDU's elevated rate (6.61 per 1,000; DUR 0.493) despite lower device utilization suggests disproportionate infection risk per catheter episode, possibly due to catheter size and aseptic technique challenges in pediatric patients.

All five NICU birth-weight strata exceeded the NHSN VLBW NICU CLABSI benchmark of 11.0 per 1,000 CL-days [13]. The ELBW rate of 37.74 per 1,000 is consistent with published Indian NICU data documenting rates of 22-47 per 1,000 CL-days [19], reflecting immature immune function, prolonged central venous access, fragile skin, and high nurse-to-patient workload [14]. The paradoxically high LBW (1,001-1,500 g) rate (25.42 per 1,000; CL DUR 0.113) is an important and unexplained finding unique to this dataset, warranting a dedicated audit of TPN protocols, insertion practices, and dressing change compliance for this birth-weight cohort.

Hand hygiene

A mean HH compliance of 21.5% -- less than one-quarter of the WHO minimum standard of 80% [15] -- is the single most striking and modifiable finding. Published Indian ICU studies report similarly low compliance (18%-39%) [8], suggesting this is a systemic national problem. Blot et al., in a systematic review and meta-analysis, estimated that every 10% absolute improvement in HH compliance is associated with a 34% reduction in CLABSI incidence [16]. The WHO multimodal HH improvement strategy, when fully implemented, has consistently demonstrated improvements from below 30% to above 60% within 12 months [15]. At our baseline of 21.5%, achieving the WHO minimum target of 80% represents a 58.5 percentage point improvement and could theoretically eliminate the majority of our device-associated CLABSI burden.

Strengths and limitations

Strengths of this study include the use of NHSN-defined infection criteria with device-day denominators, enabling valid international rate comparisons, unit-level stratification across 14 distinct ICU and HDU types, an 18-month observation period capturing a natural pre-COVID-19 improvement and pandemic-era reversal, birth-weight-stratified NICU analysis, contemporaneous HH compliance monitoring, and the maximal utilization of all available institutional surveillance data. To our knowledge, this is one of the most granular published analyses of CAUTI and CLABSI from a government tertiary teaching hospital in northern India using device-day denominators.

Limitations of this study include its retrospective design with no patient-level data available (age, sex, comorbidities, antimicrobial therapy, or CL type or insertion site); the absence of unit-level denominators for August to November 2019; the three-month pandemic surveillance gap (April to June 2020), which represents an unquantifiable blind spot for infection events during that period; potential under-ascertainment of CLABSI in units with lower surveillance staffing; inability to verify reported events against individual microbiological culture records; and the observational study design, which precludes causal inference regarding any specific intervention or exposure. The Spearman correlation between HH compliance and CLABSI rate was underpowered with only 14 monthly pairs. Generalizability to smaller or private sector hospitals in India may be limited.

## Conclusions

This 18-month retrospective CAUTI and CLABSI surveillance study from AIIMS Rishikesh demonstrates that device-related infection rates substantially and persistently exceeded NHSN benchmarks, with a 2.64-fold worsening of CLABSI during the COVID-19 pandemic. Every unit with a sufficient denominator exceeded the NHSN 90th-percentile CLABSI benchmark. HH compliance was critically suboptimal throughout. The pre-COVID-19 improvement trajectory proves that meaningful improvement is achievable in this setting. Priority recommendations include (i) structured CLABSI prevention bundles in medicine allied ICU, CCU-4, HDU and stroke, and medicine HDU; (ii) daily catheter necessity review and CAUTI prevention protocols in RICU, pediatric medicine HDU, and neurosurgery ward; (iii) WHO five-moment multimodal HH program targeting ≥80% compliance; (iv) birth-weight-stratified neonatal CLABSI prevention bundles with dedicated root-cause analysis for the LBW stratum; (v) formal daily device utilization review rounds in all ICUs targeting Foley DUR < 0.50 and CL DUR < 0.30; and (vi) designation of HICC surveillance as protected essential patient safety infrastructure with a documented pandemic contingency plan.
